# Dynamics of Soil Organic Carbon and Microbial Biomass Carbon in Relation to Water Erosion and Tillage Erosion

**DOI:** 10.1371/journal.pone.0064059

**Published:** 2013-05-22

**Authors:** Nie Xiaojun, Zhang Jianhui, Su Zhengan

**Affiliations:** 1 Key Laboratory of Mountain Surface Processes and Ecological Regulation, Chinese Academy of Sciences, Institute of Mountain Hazards and Environment, Chinese Academy of Sciences and Ministry of Water Conservancy, Chengdu, China; 2 School of Surveying and Land Information Engineering, Henan Polytechnic University, Jiaozuo, China; University of Delaware, United States of America

## Abstract

Dynamics of soil organic carbon (SOC) are associated with soil erosion, yet there is a shortage of research concerning the relationship between soil erosion, SOC, and especially microbial biomass carbon (MBC). In this paper, we selected two typical slope landscapes including gentle and steep slopes from the Sichuan Basin, China, and used the ^137^Cs technique to determine the effects of water erosion and tillage erosion on the dynamics of SOC and MBC. Soil samples for the determination of ^137^Cs, SOC, MBC and soil particle-size fractions were collected on two types of contrasting hillslopes.^ 137^Cs data revealed that soil loss occurred at upper slope positions of the two landscapes and soil accumulation at the lower slope positions. Soil erosion rates as well as distribution patterns of the <0.002-mm clay shows that water erosion is the major process of soil redistribution in the gentle slope landscape, while tillage erosion acts as the dominant process of soil redistribution in the steep slope landscape. In gentle slope landscapes, both SOC and MBC contents increased downslope and these distribution patterns were closely linked to soil redistribution rates. In steep slope landscapes, only SOC contents increased downslope, dependent on soil redistribution. It is noticeable that MBC/SOC ratios were significantly lower in gentle slope landscapes than in steep slope landscapes, implying that water erosion has a negative effect on the microbial biomass compared with tillage erosion. It is suggested that MBC dynamics are closely associated with soil redistribution by water erosion but independent of that by tillage erosion, while SOC dynamics are influenced by soil redistribution by both water erosion and tillage erosion.

## Introduction

Soil erosion has a severe impact on soil organic carbon (SOC) pools, and as a consequence affects the soil carbon cycle. Historically, water erosion was assumed to be a major soil disturbance in agricultural slope landscapes. Numerous studies have emphasized the influences of water erosion on SOC removal and carbon sequestration in agricultural slope landscapes [1]–[6]. Since the widespread application of ^137^Cs (i.e., Caesium-137) technique to assess soil erosion in the 1990s, field evidence from different regions of the world has documented that tillage erosion (i.e. the net movement of soil downslope in response to the action of farm implements) is another major soil disturbance in agricultural slope landscapes with intensive tillage [5], [7]–[11]. The impacts of tillage erosion on within-field variability of SOC have recently been addressed [12]–[14]. For example, on steep hillslopes of the Sichuan Basin, China, there is a complete depletion of SOC at summit positions and remarkable accumulation at bottom positions after 15-yr tillage erosion [14].

Although the physical process of SOC transfer on the slope under water and tillage erosion has been well known, the SOC biochemical process (e.g. carbon mineralization), mostly linked with the carbon cycle, remains unclear in eroding landscapes. Polyakov and Lal (2004) reported that no significant differences in SOC mineralization exist between the eroding and control soils, but there is a 26% higher CO_2_-efflux in the deposited soils compared with those without deposition [15]. In contrast, a recent study conducted by Hemelryck et al. (2011) shows that SOC mineralization in soils with deposition does not apparently differ from soils without deposition [16]. Van Oost et al. (2007) concluded that SOC mineralization associated with transport are relatively minor and that most deposited carbon is effectively preserved [17].

Indeed, it is difficult to make clear the relationship between erosion and SOC mineralization without considering the type of erosion event and soil microbial conditions. In fact, soil microbes participate in all of the biochemical process of SOC, thus microbial biomass affects the emission of CO_2_ under soil erosion. Soil microbial biomass carbon (MBC) generally making up 1–5% of total SOC [18], acts as the most active component in the biochemical process of SOC turnover [19] and responds more rapidly to soil disturbance than does SOC [20]. Relatively, few studies have dealt with relationships between MBC dynamics and soil erosion [21]. In the process of water erosion, selective entrainment of fine soil particles is controlled by the transport capacity of the overland flow [22], and the labile SOC fraction adhered to fine soil particles is easily mineralized [23]. In contrast, tillage erosion unselectively moves the soil downslope [14], [24], with no transport-related mineralization of SOC in the process of tillage erosion [25]. Given different mechanisms of soil transport, it would be expected that the two different processes of soil erosion may exert different impacts on MBC and the resultant SOC mineralization.

Caesium-137 (^137^Cs) is a well-established tracer of soil erosion [26]. By comparing ^137^Cs inventories of the study sites with those of reference sites, one can determine whether erosion (less ^137^Cs in study sites than in reference sites) or deposition (more ^137^Cs in study sites than in reference sites) has occurred. The relationships among patterns of soil erosion, SOC and some physio-chemical properties have been established for a number of fields and small agricultural watersheds in different climate zones [5], [27]–[29], showing that this method is a valuable tool to investigate the erosion-carbon relationship. In this study, we use the same methodology to evaluate the relationship between erosion and carbon dynamics on cultivated slopes of the Sichuan Basin, China, where both water erosion and tillage erosion have occurred.

This study aims to (1) compare the difference in soil erosion between the two different slope landscapes (i.e., gentle and steep slopes), (2) examine spatial patterns of SOC and MBC distribution over eroded slopes, and (3) elucidate effects of water erosion and tillage erosion on the dynamics of SOC and MBC.

## Materials and Methods

### Study Area

The study area was located in Jianyang County of the Sichuan Basin, southwestern China (30°04′28″–30°39′00″N, 104°11′34″–104°53′36″E). This region is typical of the hilly areas of Sichuan (400–587 m above sea level) and has a humid subtropical climate (mean annual temperature 17°C; mean annual rainfall of 872 mm with 90% of precipitation between May and October). Most of the hillslopes have been dissected into slope segments with different slope gradients during previous long-term agricultural practices to minimize soil erosion and optimize farming operations. With the hoe, a predominant tillage implement in this area, one major tillage operation per year always starts at the bottom of the slope and moves upslope step by step, but at every step the tillage direction is always downslope (i.e., always pulling down). Due to long-term intensive hoeing, those in the upper parts of the slope are characterized by a thin soil layer, underlain by bedrock, whereas deeper soils are found in the lower parts. The soils in the study area, derived from purple mudstone and sandstone of Jurassic Age, are classified as Orthic Regosols in the FAO soil taxonomy. Soil texture is clay loam (27% clay, 29% silt, 44% sand), containing 1.0–2.5% organic matter and with a pH of 7.9–8.2. Crop rotation involves [wheat (*Triticum aestivum* L.), maize (*Zea mays* L.)/sweet potato (*Ipomoea batatas*)] and fertilizers (N: 330 kg ha^−1^ year^−1^ as urea and ammonium bicarbonate, P_2_O_5_∶166 kg ha^−1^ year^−1^ as superphosphate) are uniformly applied on the cultivated slopes. Residue management is also uniformly on the slopes in the study area. For wheat and maize, the residues are cut by hand as low as possible and removed, retained residues with about 10-cm height above the ground are mixed into the tillage layer by manual hoeing. For sweet potato, all the residues are removed from the field.

### Soil Sampling

Soil sampling was conducted on two types of contrasting hillslopes. Two neighboring cultivated slopes with a length of 44 and 53 m and a corresponding gradient of 7% and 4%, respectively, were considered as two replicates for gentle slope landscapes (Table 1). Another two neighboring slopes with a length of 17 and 22 m and a corresponding slope gradient of 26% and 19%, respectively, were considered as two replicates for steep slope landscapes (Table 1). Samples were collected using a soil corer (6.8-cm diameter) along the downslope transect at 10-m intervals on the two gentle slopes and at 5-m intervals on the two steep slopes. For steep slope landscapes, there were 4, 1 and 4 samples, respectively, in the upper, middle and lower positions. For gentle slope landscapes, 4, 3 and 4 samples were collected in the upper, middle and lower positions, respectively. The coordinates of each sampling point as well as elevation were measured using a survey-grade Differential Global Positioning System (DGPS).

**Table 1:** Total soil erosion rates, tillage erosion rates and water erosion rates.

	Gentle slope landscape		Steep slope landscape	
	4%-slope	7%-slope	19%-slope	26%-slope
Total length (m)	53	44	22	17
Sample number	6	5	5	4
Total soil erosion rate (t ha^−1^ yr^−1^)	24.41	28.01	39.77	44.83
Tillage erosion rate (t ha^−1^ yr^−1^)	6.96	9.29	26.16	39.67
Percentage of total erosion (%)	29	33	66	88
Water erosion rate (t ha^−1^ yr^−1^)	17.45	18.72	13.61	5.16
Percentage of total erosion (%)	71	67	34	12

For each sampling point, five soil cores were collected. Two cores for the determination of ^137^Cs inventory were taken to the bedrock (depths range from 20 to 40 cm depending on thickness of total soil layer at different slope positions) and were then bulked to make a composite sample. In the context of this study, soil depth reached a maximum of 40 cm, and therefore sampling to this depth has all ^137^Cs in soil profiles contained in samples. Another three core samples for the determination of SOC, MBC, and soil particle-size fractions were collected from the till layer (0–20 cm). One of major considerations for this is that soil redistribution by water and tillage happened mainly in the till layer. Another is that soil microbes are the most active in the till layer where a large portion of organic matter such as crop residues are concentrated, thus the effects of soil erosion on the biochemical process of SOC can be expected to be dominant. After the three soil cores were taken, they were mixed into a composite sample and then divided into two subsamples. One was sieved (2-mm) and immediately stored at 4°C in plastic bags loosely tied to ensure sufficient aeration and to prevent moisture loss until MBC analysis. The other was air-dried and sieved (2-mm) for analyses of SOC and particle-size fractions.

### Laboratory Analysis

Soil samples for ^137^Cs determination were air-dried, crushed and passed through a 2-mm mesh sieve to remove gravels. Samples of the <2 mm particle-size fraction were packed into plastic (PVC) beakers with a 320-cm^3^ volume, and ^137^Cs activity was measured using a hyperpure lithium-drifted germanium detector (HpC –40% efficiency) coupled with a Nuclear Data 6700 multichanel γ-ray spectrophotometer. Caesium-137 was detected at 662 KeV, and count time for each sample ranged from 40,000 to 60,000 s, providing a measurement precision of ±5%. The contents of ^137^Cs were originally expressed as a unit mass basis (Bq kg^−1^) and were then converted into an area basis (Bq m^−2^) using the total weight of the bulked core sample and the cross-sectional area of the sampling device. Soil bulk densities (kg m^−3^) were determined using oven-dried weight and sample volume.

SOC was determined using wet oxidation with K_2_Cr_2_O_7_ and soil particle-size fractions were determined by pipette method following H_2_O_2_ treatment to destroy organic matter and dispersion of soil suspensions in Na-hexametaphosphate [30]. MBC was determined by fumigation with ethanol-free CHCl_3_ and extraction with K_2_SO_4_ [31]. Measurement precision for SOC, MBC, and soil particle-size fractions is ±2%, ±4%, and ±2%, respectively.

### Calculation of Soil Erosion Rates

The application of the ^137^Cs technique and tillage erosion models provides a new perspective to assess the contribution of water and tillage to total soil erosion [5], [9]–[11]. For an eroding site where a total ^137^Cs inventory *A* (Bq m^−2^) is less than the reference inventory *A*_0_ (Bq m^−2^) at year *t* (date), soil erosion rates can be expressed as [32]:
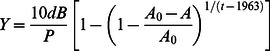
(1)where *Y* is the soil erosion rate (t ha^−1^ yr^−1^), *d* the sampling depth (m), *B* the bulk density of soil (kg m^−3^) and *P* the particle size correction factor (assumed to be *P* = 1.0). According to the previous study conducted in the study area [5], a local ^137^Cs reference inventory was calculated as 1318 Bq m^−2^ with radioactivity decay corrected to the year 2008.

Based on a large number of experimental observations conducted previously in the study area, an empirical model of tillage erosion has been well established by Zhang et al. (2004) [33]. We adopted the model in this study because of similar terrain attributes such as the shape (linear), ranges of gradient and slope length, and even similar soil properties. In the empirical model, tillage erosion rates increases with increasing slope gradient and is inversely proportional to the slope length, expressed as:
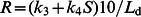
(2)where *R* is the tillage erosion rate (t ha^−1^ yr^−1^), *k*_3_ and *k*_4_ are the soil transport coefficient (kg m^−1^ yr^−1^), *S* is the slope gradient (m m^−1^) and *L*_d_ is the downslope parcel length (m). Tillage transport coefficients in the study area reach 30.72 and 141.28 kg m^–1^ yr^–1^, respectively, for *k*_3_ and *k*_4_ [33]. Water erosion rates were obtained by the differences between total soil erosion rates derived from ^137^Cs data and tillage erosion rates [5], [11].

### Data Analysis

Statistical analyses were carried out using SPSS 11.0 for Windows version (SPSS Inc., US, 2002). Linear regression analysis was used to test the correlations between ^137^Cs, SOC and MBC. One-way analysis of variance was used to test the significance of differences in MBC/SOC ratios between gentle and steep slope landscapes, and between upper and lower positions of the two slope landscapes.

### Ethics Statement

In this study, soil sampling and sample determinations conducted were permitted by the local government (i.e. Jianyang County People’s Government). We also obtained a permission from the local government for reporting research results to the public.

## Results

### Soil Redistribution

**Figure 1:**
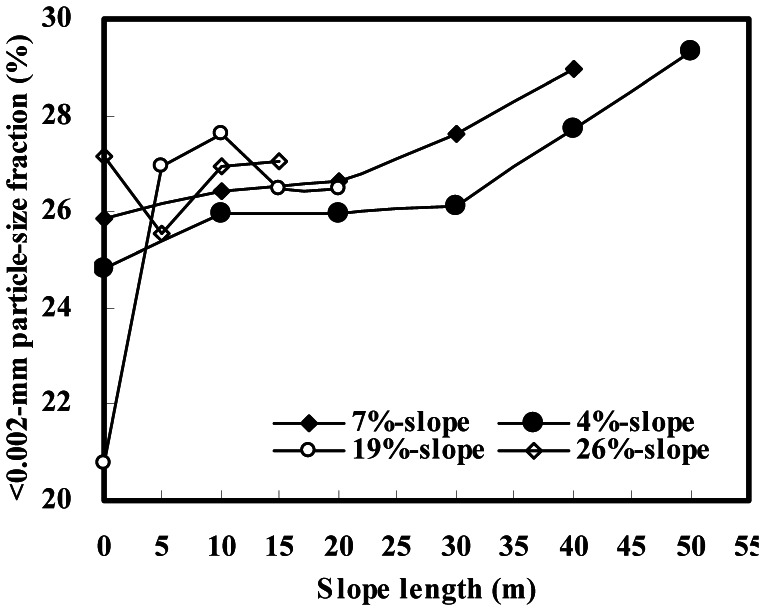
Distribution of the <0.002-mm particle-size fraction on eroded slopes.

For steep slope landscapes, tillage erosion rates were 26.16 and 39.67 t ha^−1^ yr^−1^ and accounted for 66% and 88% of total erosion rates for the 19%- and 26%-slopes, respectively (Table 1). For gentle slope landscapes, tillage erosion rates were 9.29 and 6.96 t ha^−1^ yr^−1^ and contributed only 33% and 29% to total erosion rates for the 7%- and 4%-slopes, respectively (Table 1). The results indicate that tillage erosion dominates the process of soil redistribution in steep slope landscapes, whereas water erosion is a major process of soil redistribution in gentle slope landscapes. Differences in dominant processes of soil erosion between the two slope landscapes were also supported by the distribution of the <0.002-mm particle-size fraction on the eroded slopes. The fine particle fraction gradually increased downslope on each gentle slope, but such a trend of the fine particle was not observed on the two steep slopes (Figure 1). This result suggests that soil fine particles are preferentially transported downslope mainly by water in gentle slope landscapes, while the soils are unselectively or entirely transported downslope due to the dominant process of tillage erosion in steep slope landscapes.

**Figure 2:**
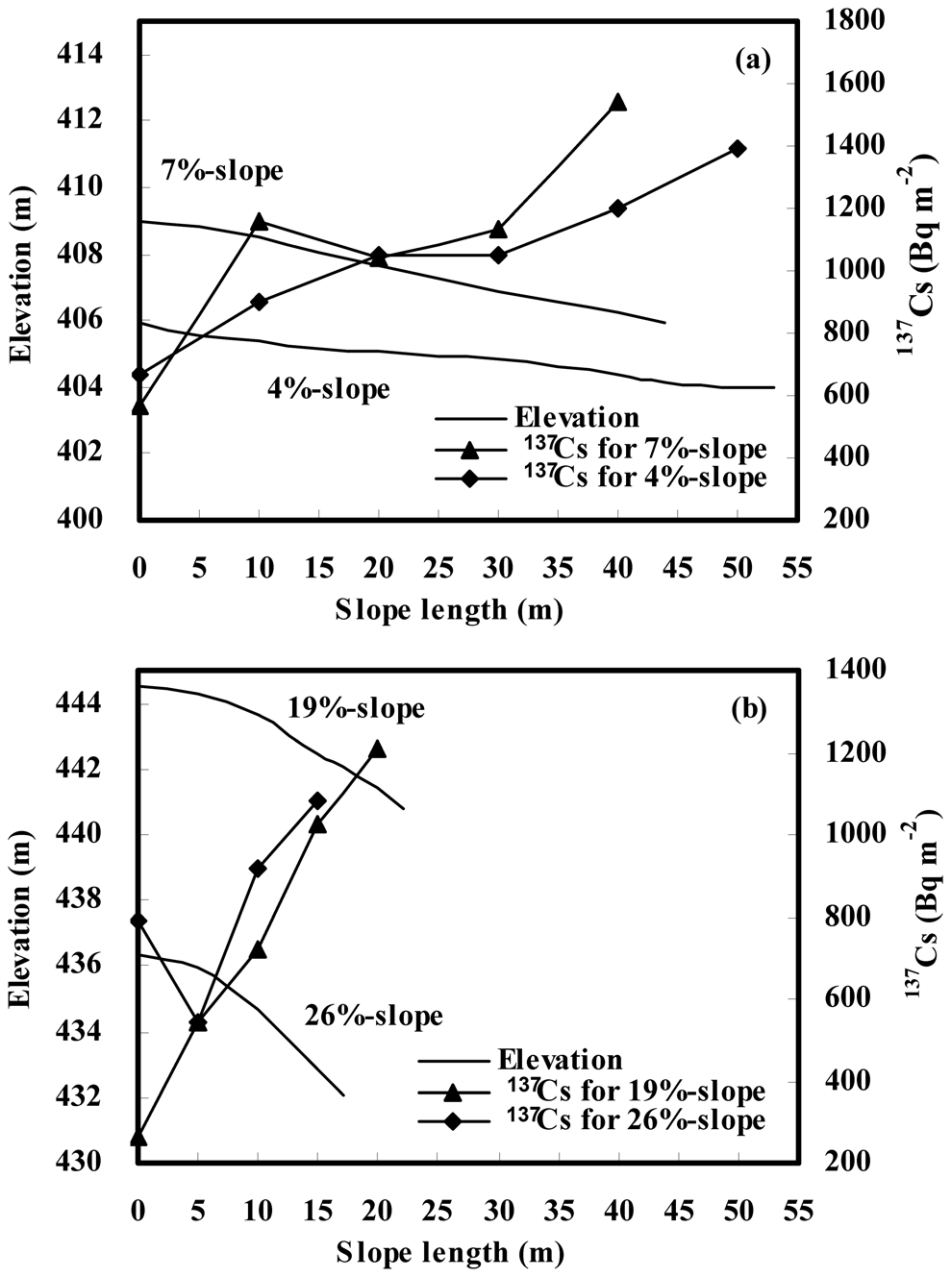
Distribution of ^137^Cs inventories over eroded slopes. (a) gentle slope landscape; (b) steep slope landscape.

Figure 2 shows a similar ^137^Cs distribution pattern in gentle and steep slope landscapes, i.e., ^137^Cs inventory increased along the downslope transect. For gentle slope landscapes, ^137^Cs inventory increased from 861 to 1336 Bq m^−2^ and 786 to 1289 Bq m^−2^ from the upper to lower positions of the 7%- and 4%-slopes, respectively (Table 2). For steep slope landscapes, ^137^Cs inventory ranged from lows of 402 and 668 Bq m^−2^ at upper slope positions to highs of 1179 and 1002 Bq m^−2^ at lower slope positions of the 19%- and 26%-slopes, respectively (Table 2). The results suggest that soil loss occurs at upper slope positions and soil accumulation at lower slope positions.

**Table 2:** Descriptive statistics of ^137^Cs inventories, SOC and MBC contents in different positions for gentle and steep slope landscapes.

Gentle slope landscape	4%-slope			7%-slope		
Slope position	Upper	Middle	Lower	Upper	Middle♀	Lower
Slope length	0–10	20–30	30–53	0–10	20	30–44
Sample number	2	2	2	2	1	2
Mean ^137^Cs (Bq m^−2^)	786	1051	1296	861	1040	1336
Mean SOC (g kg ^−1^)	9.33	9.84	12.42	9.20	9.39	12.99
Mean MBC (mg kg^−1^)	168.78	202.67	221.13	182.13	180.95	217.80
Mean MBC/SOC ratio	1.81×10^−2^	2.06×10^−2^	1.78×10^−2^	1.99×10^−2^	1.93×10^−2^	1.69×10^−2^
**Steep slope landscape**	**19%-slope**			**26%-slope**		
Slope position	Upper	Middle♀	Lower	Upper	Lower	
Slope length	0–5	10	10–22	0–10	10–17	
Sample number	2	1	2	2	2	
Mean ^137^Cs (Bq m^−2^)	402	723	1117	668	1002	
Mean SOC (g kg ^−1^)	7.33	8.49	10.15	9.33	11.82	
Mean MBC (mg kg^−1^)	242.45	308.44	234.41	257.09	273.62	
Mean MBC/SOC ratio	3.28×10^−2^	3.63×10^−2^	2.32×10^−2^	2.83×10^−2^	2.32×10^−2^	

♀ single value in the position.

### SOC and MBC Dynamics

**Figure 3:**
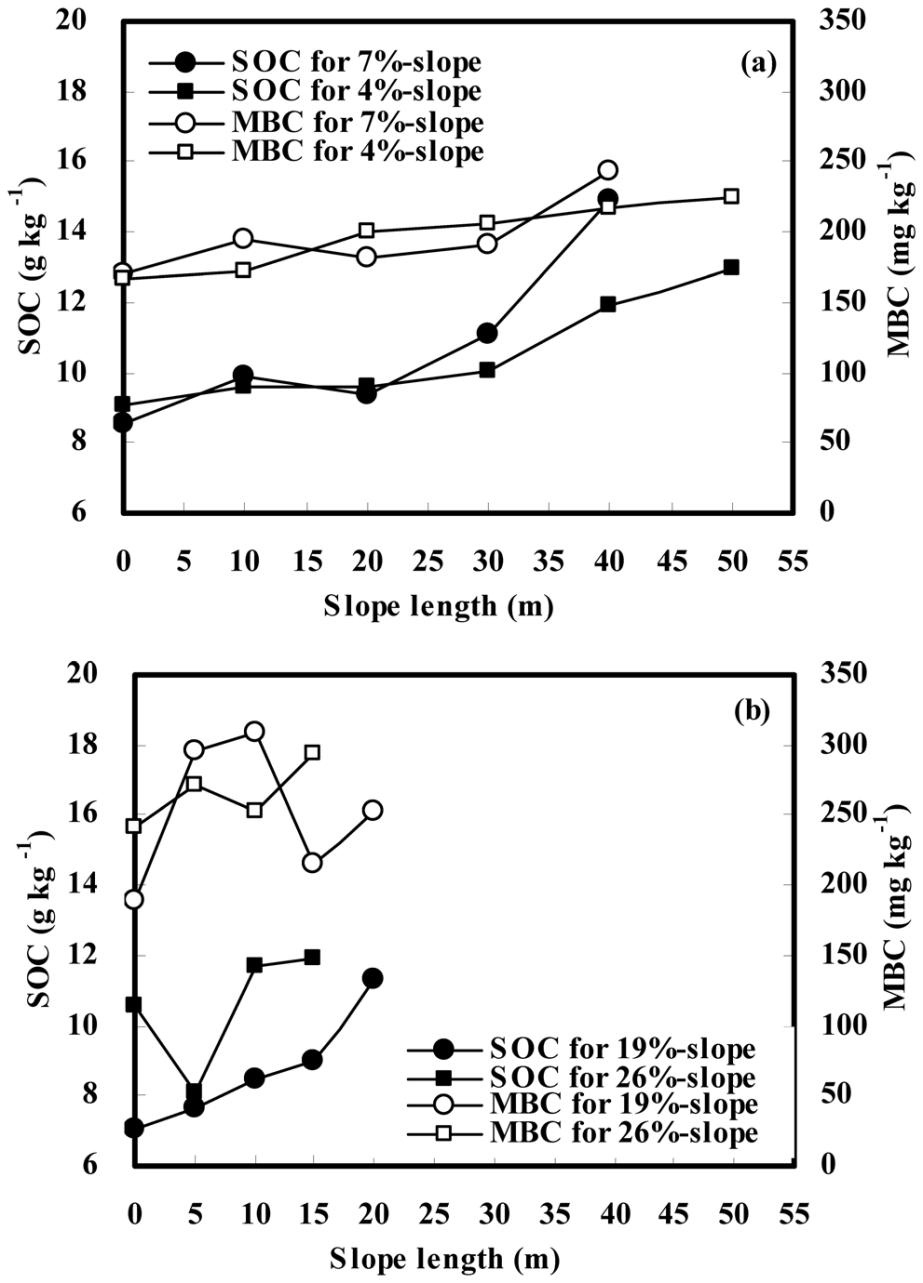
Distribution of SOC and MBC contents over eroded slopes. (a) gentle slope landscape; (b) steep slope landscape.

**Figure 4:**
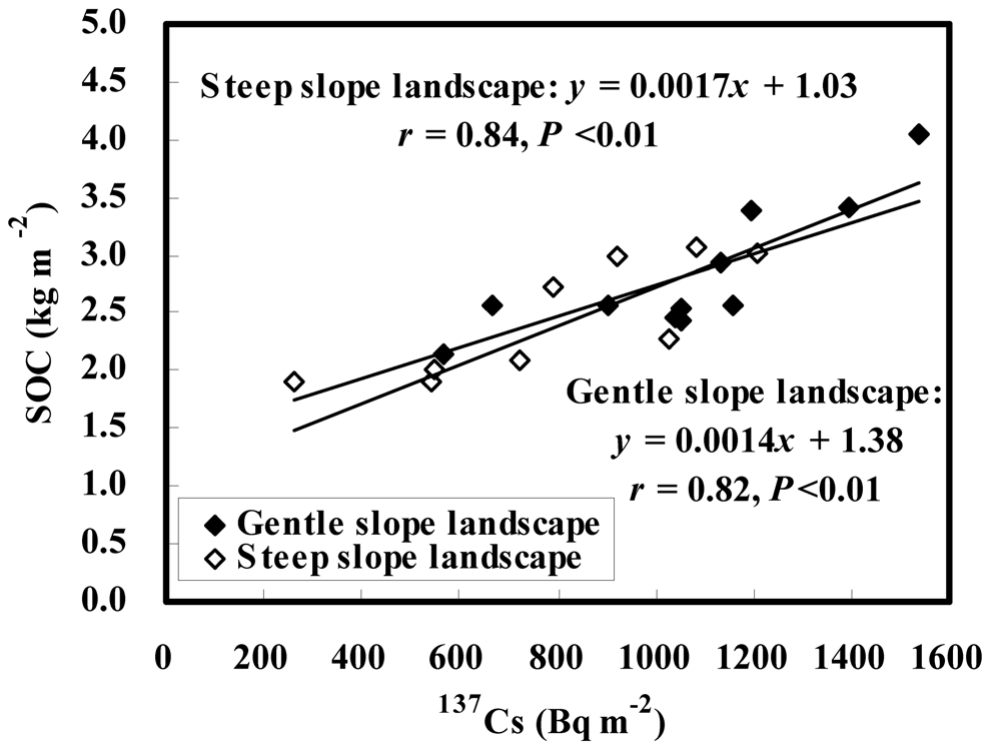
SOC contents vs. ^137^Cs inventories in gentle and steep slope landscapes.

**Figure 5:**
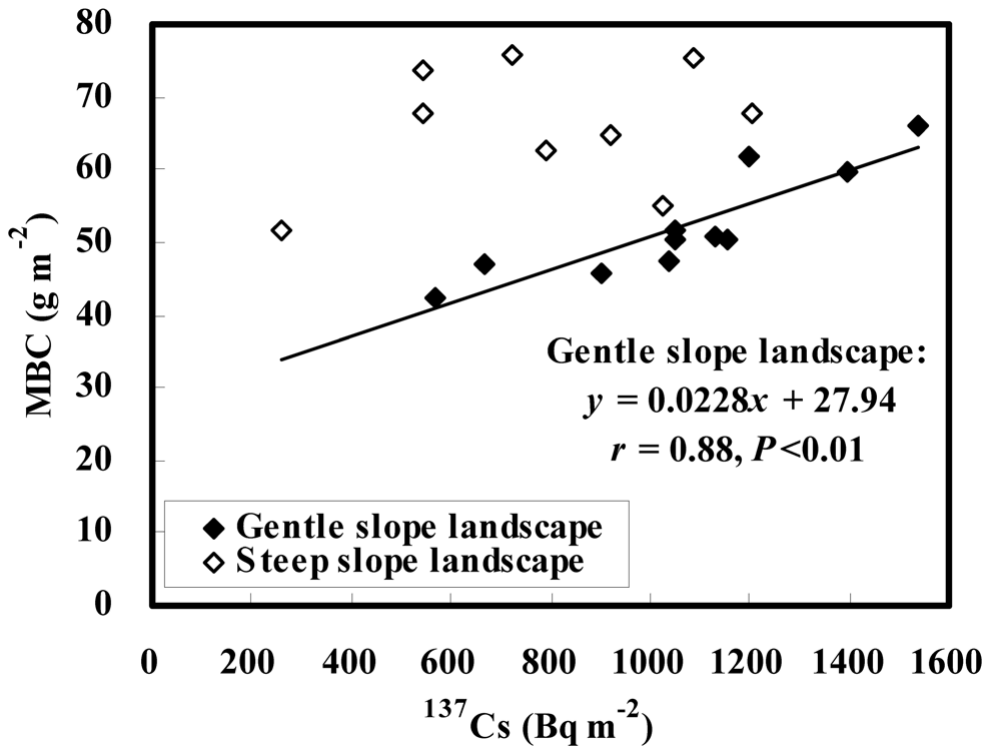
MBC contents vs. ^137^Cs inventories in gentle and steep slope landscapes.

In gentle slope landscapes, both SOC and MBC contents increased downslope in a roughly consecutive increment (Figure 3a). SOC contents averaged 12.99 and 12.42 g kg^−1^ at lower slope positions of the 7%- and 4%-slopes with an increase of 44% and 31%, respectively, compared with those at respective upper slope positions (Table 2). From the upper to lower slope positions, MBC contents changed from 182.13 to 217.80 mg kg^−1^ with an increase of 20% on the 7%-slope, and from 168.78 to 221.13 mg kg^−1^ with an increase of 31% on the 4%-slope (Table 2). These patterns are in agreement with the distribution pattern in ^137^Cs inventory that mirrors soil redistribution rates on the two gentle slopes. Correlation analysis also showed that inventories of SOC (kg m^−2^) and MBC (g m^−2^) were significantly correlated with ^137^Cs inventory (Bq m^−2^) in the landscape (*r* = 0.82, *P*<0.01 and *r* = 0.88, *P*<0.01, respectively, for SOC and MBC; see Figures 4, 5). As a consequence, it is clear that the dynamics of SOC and MBC are closely associated with soil redistribution in gentle slope landscapes.

Different distribution patterns between SOC and MBC contents were observed in steep slope landscapes, showing a rough increase in SOC content but a discrete pattern in MBC content along the downslope transect (Figure 3b). From the upper to lower slope positions, SOC contents changed from 7.33 to 10.15 g kg^−1^ with an increase of 38% on the 19%-slope, and 9.33 to 11.82 g kg^−1^ with an increase of 27% on the 26%-slope (Table 2). An increase in MBC content was observed only at middle positions of the 19%-slope, but little change in MBC between the upper and lower positions of each steep slope (Table 2). The distribution pattern in SOC content is consistent with ^137^Cs inventory on each steep slope. A significant correlation (*r* = 0.84, *P*<0.05) between SOC (kg m^−2^) and ^137^Cs (Bq m^−2^) was also found in steep slope landscapes (Figure 4). However, MBC distribution could not be explained by ^137^Cs data on each steep slope, as a result of no correlation (*r* = 0.25, *P*>0.05) between MBC (g m^−2^) and ^137^Cs (Bq m^−2^) in steep slope landscapes (Figure 5). Thus it is suggested that SOC dynamics are remarkably impacted by soil redistribution in steep slope landscapes, whereas MBC dynamics are independent of soil redistribution in the landscape. The close relationship between the <0.002-mm fine particle and MBC contents in gentle slope landscapes dominated by water erosion (*r* = 0.86–0.93, *P* = 0.02–0.03; data not shown) also showed that the microbial biomass decreased with decreasing soil fine particles under water erosion. However, no significant correlations between the two parameters (*r* = −0.22–0.79, *P* = 0.11–0.78; data not shown) were found in steep slope landscapes, implying that the microbial biomass is scarcely impacted by tillage erosion which produces unselective soil particle movement on the slope.

### Relationship between MBC and SOC

**Figure 6:**
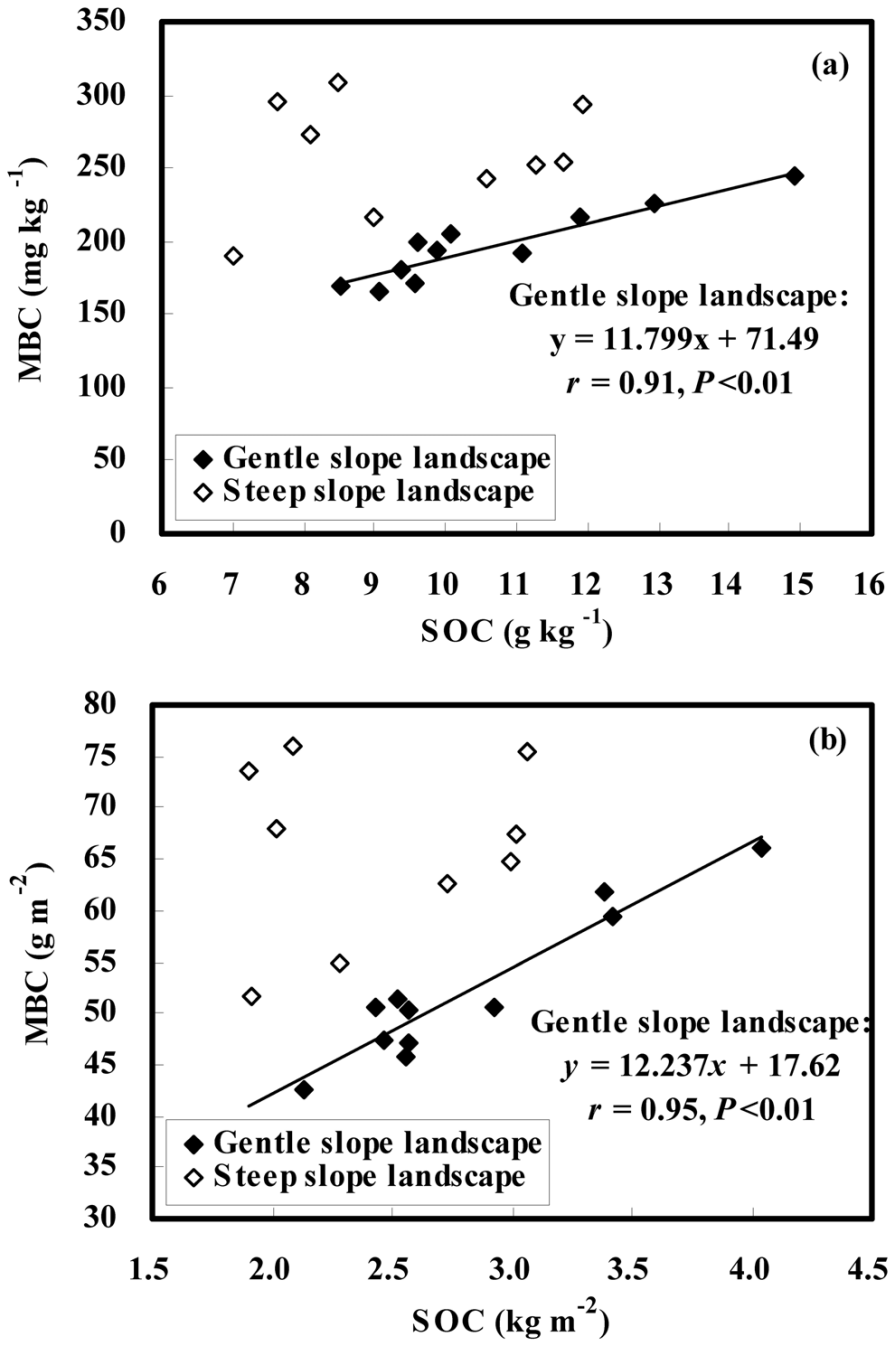
Relationship between MBC and SOC in gentle and steep slope landscapes. (a) mass basis; (b) area basis.

A close correlation (*r* = 0.91, *P*<0.01) between MBC and SOC contents (mass basis) was found in gentle slope landscapes, whereas the two were not correlated in steep slope landscapes (*r* = 0.18, *P*>0.05) (Figure 6a). When the contents of SOC and MBC were expressed on a unit area basis (kg m^−2^ and g m^−2^, respectively), a similar correlation (*r* = 0.95, *P*<0.01) between the two variables was also found in gentle slope landscapes, but there was still no correlation (*r* = 0.18, *P*>0.05) in steep slope landscapes (Figure 6b). In this study, additional information on the relationship between MBC and SOC was also obtained from changes in the ratio of MBC (g kg^−1^) to SOC (g kg^−1^). The MBC/SOC ratio was slightly greater at upper positions than at lower positions of each slope (Table 2). However, there were no statistically significant differences in MBC/SOC ratios between upper and lower positions of the two slope landscapes (*P*>0.05). The results indicate the difference in the MBC fraction per unit of SOC between the upper and lower positions was insignificant in the landscape. In terms of the whole slope, the MBC/SOC ratio in steep slope landscapes averaged 2.77×10^−2^, and was significantly greater than that (mean 1.87×10^−2^) in gentle slope landscapes (*P*<0.05). This showed a smaller MBC fraction per unit of SOC in gentle slope landscapes than in steep slope landscapes, suggesting that MBC is influenced by different processes of soil redistribution in the landscape.

## Discussion

The distribution pattern of SOC content was consistent with redistribution patterns of ^137^Cs inventory in the two slope landscapes. The result agrees with previous studies [5], [11], [28], further confirming a close relationship between SOC translocation and soil redistribution by water and tillage. The MBC distribution pattern was in agreement with soil redistribution in gentle slope landscapes but independent of soil redistribution in steep slope landscapes. This is attributed to impacts of water-induced soil redistribution on SOC and MBC in gentle slope landscapes, and impacts of tillage-induced soil redistribution in steep slope landscapes. The difference in the relationship between MBC and SOC under the disturbances of water and tillage erosion differed from the results of previous studies. A close correlation between MBC and SOC has been reported under various anthropic disturbances such as plant cover, fertilization, organic amendments, and tillage [34]–[37]. It is, therefore, that the dominant erosion process should be first considered when one assesses the dynamics of MBC and SOC in eroded slope landscapes.

In the two slope landscapes, lower SOC contents were found at upper slope positions with soil loss than at lower slope positions with soil accumulation. The result indicates the consistent physical process of SOC depletion in upper slope positions and SOC accumulation in lower slope positions regardless of water- or tillage-induced soil transport. However, changes in MBC contents were inconsistent between upper and lower positions in the two slope landscapes. On the two gentle slopes, MBC contents were apparently 20% and 31% higher in lower slope positions than upper slope positions, thus indicating that the physical transfer of MBC is related to soil redistribution. On the two steep slopes, however, there was little difference in MBC content between upper and lower positions, suggesting a negligible impact of soil redistribution on the physical dynamics of MBC. This similarity of MBC content between the two positions in steep slope landscapes could be attributed to unselective removal and the dilution effect due to the incorporation of the soil from deeper layers (e.g. subsoils) into the till layer by tillage (i.e. MBC contents in the newly formed surface soil decrease compared to the original surface soil, due to poor MBC in the subsoils) [14], [17]. In the study area, the farmers have to till into subsoils, parent material, or even bedrocks to offset soil loss in the till layer. The soil translocated from the upslope moves entirely downslope and subsequently deposits at lower slope positions under consecutive tillage operations. Consequently, little impact on soil microbial biomass was present in the till layer in the line of slope, as a result of the redistribution of low fertility soils. No statistically significant difference (*P*>0.05) in MBC/SOC ratios between upper and lower positions in the two slope landscapes showed that the erosion-induced SOC depletion did not apparently decrease the ratio of MBC to SOC, and the deposition-induced SOC accumulation did not obviously increase this ratio either.

The MBC/SOC ratio represents the contribution of microbial biomass to organic carbon in soil [38], and is a more useful assessment index for soil health than either MBC or SOC [39]. The lower mean of MBC/SOC ratios in gentle slope landscapes than in steep slope landscapes (*P*<0.05) shows a smaller MBC contribution to SOC pools in gentle slope landscapes. This also implies that water erosion with selective removal of soil fine particles has a more severe disturbance to soil microbial biomass than tillage erosion with unselective soil movement. The difference in MBC/SOC between the two slope landscapes is probably associated with different responses of microbial biomass to distinctive changes in soil fine particles. Insam et al. (1989) reported that the clay content of soil has an important influence on the variance in MBC/SOC [40]. It is well known that soil clay (i.e. the <0.002-mm fine particle) has a strong adhesion to organic matter due to its large surface area, and thus is regarded as the mineral binding agents to form soil aggregates. In the process of water erosion, soil clay is preferentially removed by runoff, thus disrupting soil aggregates [5], [41]. Accordingly, microbes incorporated into initial soil aggregates die due to their exposure to an unprotected circumstance [23], which results in a decline in soil microbial biomass. It should be noted that the same tillage operation including tillage intensity (one time per year) and tillage tool (hoe) is performed in the two slope landscapes, although tillage reduces aggregate stability by exposing the encapsulated SOC binding agents to mineralization [42]. As a result, impacts of aggregate breakdown by tillage would be similar between the two slope landscapes. In this sense, the difference in soil microbial biomass between the two landscapes resulted from different processes of soil redistribution (dominated by water erosion or by tillage erosion), excluding direct impacts of aggregate breakdown due to tillage operations.

### Conclusion

In water-eroded slope landscapes, both SOC and MBC contents showed a significant correlation with soil redistribution rates. In tillage-eroded slope landscapes, SOC contents also exhibited a similar relationship, whereas MBC contents did not reveal any clear pattern. The MBC/SOC ratio was lower on the water-eroded slope than on the tillage-eroded slope, while the contribution of MBC to SOC pools was similar between the erosion-induced SOC depletion and SOC accumulation sites irrespective of landscape types. It is clear that water erosion has a severe disturbance to soil microbial biomass compared with tillage erosion. We conclude that SOC dynamics are associated closely with soil redistribution by water erosion and by tillage erosion as well, while MBC dynamics are significantly influenced by water-induced soil redistribution, but independent of tillage-induced soil redistribution.
